# Does Climate Change Cause an Upsurge in Food Prices?

**DOI:** 10.3390/foods13010154

**Published:** 2024-01-02

**Authors:** Sinan Erdogan, Mustafa Tevfik Kartal, Ugur Korkut Pata

**Affiliations:** 1 Department of Economics, Hatay Mustafa Kemal University, Hatay 31060, Türkiye; phderdogan@gmail.com; 2 Clinic of Economics, Azerbaijan State University of Economics (UNEC), Baku AZ1001, Azerbaijan; 3 Borsa Istanbul Strategic Planning, Financial Reporting, and Investor Relations Directorate, İstanbul 34467, Türkiye; mustafatevfikkartal@gmail.com; 4 Adnan Kassar School of Business, Lebanese American University, Beirut 1102-2801, Lebanon; 5 Department of Banking and Finance, European University of Lefke, Lefke 99010, Northern Cyprus, Türkiye; 6 Department of Economics, Osmaniye Korkut Ata University, Osmaniye 80000, Türkiye

**Keywords:** food prices, climate change, heatflation, Nigeria, non-linear methods

## Abstract

Climate change is the reason behind most contemporary economic problems. The rising inflationary pressures in the food sector are one of these problems, and stable food prices are a necessity for economic development and social cohesion in societies. Therefore, this study analyzes the relationship between food prices and climate change in Nigeria by using various non-linear and quantile-based methods and data from 2008m5 to 2020m12. The empirical findings indicate that (i) there is a time- and frequency-based dependence between food prices and some explanatory variables, including climate change (i.e., temperature). (ii) At higher quantiles, temperature, oil prices, food exports, monetary expansion, global food prices, agricultural prices, and fertilizer prices stimulate food prices. (iii) The increase in food prices due to the rise in temperature and the difficulties in agriculture indicate that the heatflation phenomenon is present in Nigeria. The evidence outlines that Nigerian decisionmakers should adopt a national food security policy that considers environmental, agricultural, and monetary factors to stabilize food prices.

## 1. Introduction

Until the Industrial Revolution, humanity was caught in a vicious circle of population growth and food production. An increase in the amount of food due to population decline made it possible to supply the larger population with food. The global economy has therefore experienced population growth. However, population growth led to an increase in food demand, and the existing production possibilities were not sufficient to supply the larger populations. Therefore, the risk of scarcity increased, and the periods of scarcity led to a decline in population. This decrease in population led to a decrease in food demand, which, in turn, led to an increase in the amount of available food. A similar cycle also took place with smaller technological advances in agriculture. Thus, humanity has lost tons of its advantages related to food quantities due to population growth. This vicious cycle is known as the “Malthusian trap” [1,2]. Technological progress fostered by the Industrial Revolution has ended the Malthusian trap, and humanity has experienced almost three centuries of plenitude.

The situation has begun to change since the second half of the 20th century. Overall, 30% of the world’s population is currently affected by food insecurity, and more than 800 million people worldwide suffer from hunger [3]. The current trend of food consumption has put pressure on sustainable food supply chains as well. The world is devoid of almost one third of the total food produced due to food loss or waste [4], meaning that food prices can become more volatile because of losses. Even worse, fluctuations and spikes in food prices can historically lead to a rise in social unrest, impacting the nature of economic measurements and data. For instance, the global economy has witnessed two major events of social unrest due to booms in food prices. The first boom in food prices occurred in 2007–2008 and caused food riots across developing countries in Africa, Europe, America, and Asia, while the second boom in food prices occurred at the end of 2010 and profoundly impacted the Sub-Saharan Africa region [5]. Therefore, the topic of food is increasingly becoming a significant policy subject for decisionmakers across the globe for ensuring a sustainable supply of food and green policy-making decisions [6], and the topic of food has significance for achieving inclusive economic development in emerging countries. 

**Figure 1:**
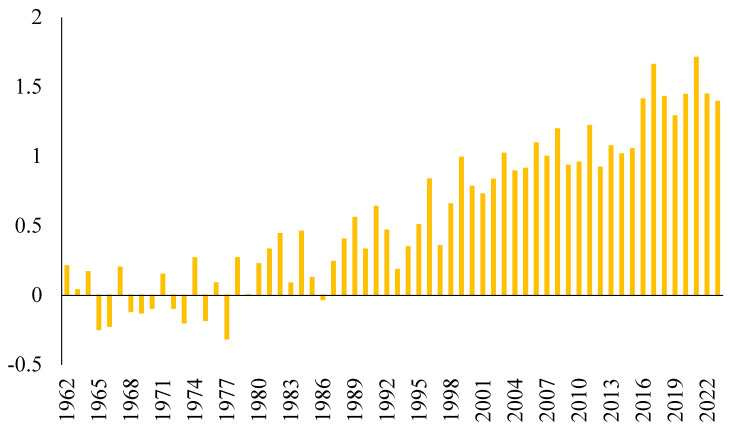
The change trend regarding the world’s surface temperature. Source: World Bank [12].

Indeed, climate change poses a significant risk to the global food supply, and the effects of global warming and climate change have worsened. Figure 1 shows the historical trend regarding global surface temperature and the temperature gap for 1961 to 2022. It can be said that the realized temperature values have risen over the years, and the temperature gap has historically been growing and continues to do so. In this regard, one can say that the ecological consequences of economic activities are becoming increasingly clear [7]. Researchers have emphasized that a typical consequence of climate change is a decline in agricultural productivity and the crop yields of key agricultural commodities (e.g., corn, soybeans, wheat, rice, sorghum, cotton, and oats) which are vital for food supply and nutrition [8]. In addition, food production is heavily dependent on water and soil quality. Unfortunately, global warming and the concentrations of greenhouse gas emissions are changing the rainfall regime, reducing sustainable water supply and degrading soil quality while increasing evaporation due to high temperatures. This process can lead to the depletion of surface water and groundwater sources for food production and cause droughts that threaten food supplies worldwide [9,10]. Therefore, achieving sustainable food production is one of the most significant challenges of this century [11]. 

Extreme heat can also increase crop failures because extreme heat waves can distort the life cycle of plants by disrupting key periods of plant growth [13]. Falling crop yields and agricultural products due to extreme heat can lead to shortages and an increase in food prices. This phenomenon is referred to in the economic literature as “heatflation” [14,15]. Heatflation is one of the biggest threats to ensuring food security and price stability in global economics, and the impacts of heatflation could be even more severe in climatically vulnerable regions. In fact, heatflation could be even more severe if population growth continues to trend upwards. Arora [16] points out that the global population growth rate is higher than the growth in crop yields per hectare. The Food and Agriculture Organization of the United Nations [17] states that the global food demand in 2050 will be 60% higher than in 2006 due to population and wealth growth. There is also the risk of a decline in global agricultural yields of between 5% and 60% by 2100 if current climate change continues. In this respect, the impact of heatflation on food supplies could be greater than expected. Therefore, heatflation is one of the principal obstacles to achieving the “Zero Hunger” goal of the SDGs.

Nigeria is the largest economy in Sub-Saharan Africa, with a GDP of almost USD 535 billion in 2022 [18]. The IMF [19] reports that Nigeria is one of the fastest-growing countries in the world, with an average economic growth of 3–6% per year. Nigeria is also one of the fastest-growing countries in terms of population growth, ranking 20th in the world [20]. However, there are several challenges for the Nigerian economy. The COVID-19 pandemic has negatively impacted the Nigerian economy in the agriculture and livestock sectors. The World Bank [21] reports that almost 58% of households engaged in food production (fishing, livestock) have suffered income losses, and the proportions of households experiencing moderate or severe food insecurity have reached record highs of 76.8% and 30.4%, respectively. In addition, 36% of Nigerian households that owned livestock were severely affected by COVID-19 in terms of access to feed (89% of affected households), access to medical supplies/medicines (79% of affected households), and access to input/output markets (82% of affected households). Overall, 17% of households that owned livestock had to sell their livestock to cope with the negative effects of the pandemic. Balana et al. [22] explain that the Russian invasion of Ukraine has increased food insecurity in Nigeria, as the country is heavily dependent on rice and wheat imports from the region.

Nigeria is also one of the countries most threatened by climate change. Bello et al. [23] reported that the global climate crisis is having a profound impact on Nigeria, increasing the country’s fragility risk. Extreme heatwaves, prolonged droughts, insufficient and erratic rainfall regimes, and floods have affected the Nigerian economy in multiple dimensions and reduced food supplies. In addition, erroneous agricultural practices degrade soil quality and reduce agricultural productivity. Idumah et al. [24] and Tajudeen et al. [25] emphasize that the impact of climate change on food production in Nigeria could increase in the coming years, as more than 80% of crop production depends on rainfall. The Notre Dame Global Adaptation Initiative [26] reports that Nigeria is the 53rd most vulnerable country and the 6th least prepared country in the world in terms of adapting to climate change. Thus, considering the factors that increase the overall demand for food (e.g., wealth and population growth), together with the factors that reduce overall food supply (e.g., conflicts and pandemics), it can be concluded that the existing incompatibilities between Nigeria’s food demand and food supply could be exacerbated by climate change. Therefore, the question of whether there is a risk of heatflation in Nigeria is a key consideration. This study aims to provide an answer to this question.

The primary objective of this study was to analyze whether there was a heatflation risk in Nigeria for the period from 2008/5 to 2020/12 by using non-linear methods (wavelet coherence (WC), quantile on quantile regression (QQ), Granger causality in quantiles (GQ), and quantile regression (QR)). It is well known that ensuring food security is one of the most important tasks of policymakers in order to maintain socio-economic stability and economic development. Therefore, this study aims to contribute to the literature in the following ways. First, to the best of our knowledge, this is the first study to investigate whether heatflation risk exists in Nigeria. Understanding the interaction mechanism between climate change and food prices will enable Nigerian policymakers to take effective climate adaptation and food security measures. Second, if policymakers know whether Nigeria is at risk of a heatflation, they can balance Nigerian food demand and supply by setting countermeasures. In this way, possible price spikes can be hindered. Third, the study involved conducting an empirical investigation by considering various time-, frequency- and quantile-based effects through the application of novel non-linear methods. The use of a set of non-linear methods allows researchers to adequately capture possible non-linearities in the data and obtain robust findings in the case of data non-normality. Thus, the effects of different variables on Nigerian food prices can be examined across time points, frequencies, and quantiles. Overall, as mentioned above, this study makes three unique contributions to the literature.

Section 2 provides an overview of the existing literature. Section 3 describes our methods in detail. Section 4 presents the results of our study. Finally, Section 5 presents the conclusions that can be drawn from this study, as well as caveats for future Nigerian policies.

## 2. Literature Review

The upward trends in global population and economic growth have increased the global food demand, while a significant number of people are being affected by food insecurity and hunger. In addition, declining crop yields due to climate change are putting significant pressure on sustainable and accessible food supplies in some regions of the world. Therefore, ensuring a balance between food demand and supply, which in turn stabilizes food prices, is a major task for policymakers. The ambitious goal of eradicating world hunger under the SDGs makes it all the more important to understand the nature of food and agricultural commodity markets. Despite their centrality to achieving the SDGs, many existing studies have ignored uncovering what drives food prices. Instead, researchers have mostly focused on the investigation of what drives food insecurity, food access, food provision, etc., and dozens of empirical papers have been written on these subjects. For instance, by conducting a regression analysis in Ghana, Adom [27] revealed that interest and exchange rates and oil prices reduce food access, while an increase in income level fosters food access. Panukhnyk et al. [28] investigated what determines food provision in Ukraine and outlined that food provision is significantly impacted by macro- and microeconomic regulations. Awad [29] examined the drivers of food insecurity in developing regions by utilizing a panel-corrected standard error method and reported that population growth, health services, and food production do not impact food insecurity, whereas political institutions, per capita income, and education drive food insecurity. Zorbas et al. [30], in a study on Australia carried out by conducting in-depth interviews, unveiled that COVID-19 has obstructed people’s abilities to accommodate to their basic living needs. Akbar et al. [31], by utilizing a binary logistic regression model for Indonesia, reported that gender, family size, age, occupation, education, income, and expenditure impact food insecurity. 

However, the literature on the drivers of food inflation remains relatively limited compared to other research subjects. One can see that only a limited number of researchers have looked at using different methods to find out what drives food prices in different areas. The general characteristics of the existing literature summarized in Table 1 can profoundly be discussed under six main themes. First, most of the former studies have mainly focused on country-specific cases and investigated the factors affecting food prices in countries with high climate and food security vulnerability (e.g., Ethiopia, Pakistan, and Nigeria). However, some studies have investigated the impact of demand- and supply-related factors on food prices (see Gilbert [32], Irz et al. [33], and Oral et al. [34]). Therefore, no conclusions can be drawn about the impact of climate events on food prices. Second, many studies in the literature have reported controversial findings on the impact of income on food prices. Normally, in a Keynesian manner, one might expect higher income levels to increase personal consumption expenditures, including food purchases, which would increase aggregate demand and prices. However, Ahsan et al. [35], Hilegebrial [36], and Rehman and Khan [37] empirically tested how income level impacts food prices by using various empirical methods. They found that an increase in income levels lowers food prices. Therefore, one can assert that the impact of income on food prices is a controversial topic in the literature. Third, some of the researchers have reported that money supply may not be systematically linked with food prices [32,36], while another group of researchers reported that an increase in the quantity of money can cause an upsurge in food prices [34,35,38,39]. Therefore, there is a controversial finding on the impact of financial conditions (e.g., money supply) on food prices as well. Fourth, the impact of energy prices on food prices is controversial. For example, the empirical findings reported by Gilbert [32] and Sujithan et al. [40] suggest that an increase in energy prices does not always lead to an increase in food prices. Fifth, Ahmed and Singla [38], Ahsan et al. [35], Hilegebrial [36] and, Oral et al. [34] suggest that there is an interdependence between world markets and national markets. Thus, fluctuations in global food markets could have an impact on national markets and prices. Sixth, linear estimation methods (e.g., Granger causality, ARDL, VECM ordinary least squares) have generally been used to uncover the drivers of food prices. However, non-linearity and non-normality in economic data are common conditions that have been considered in only a few studies (see Kartal and Depren [41]), going largely ignored in the existing literature. 

Despite the fact that Nigeria is one of the most climate change-vulnerable countries and the fact that it has a low adaptive capacity to global warming, only one study in the literature, authored by Fasanya and Olawepo [42], has examined the drivers of food prices in Nigeria. This study focuses only on financial conditions and energy prices and ignores the interlinkages between Nigerian and international food markets and supply and demand factors in Nigeria. Therefore, unlike previous studies, our study examines the supply, demand, and financial drivers of food prices in Nigeria by incorporating the effects of climate change into an analysis within the heatflation paradigm. Therefore, the main research question of this study can be expressed as follows: “how do temperature rises impact food prices in Nigeria?”. Additionally, the following hypotheses have been tested by considering the theoretical debates in the literature to fill an existing gap in the literature [14,34,41,43,44]:

**Hypothesis 1 (H1).**  *Cereal yield has a negative impact on food prices*.

**Hypothesis 2 (H2).**  *Crop production has a negative impact on food prices*.

**Hypothesis 3 (H3).**  *Food exports have a positive impact on food prices.*

**Hypothesis 4 (H4).**  *The food production index has a negative impact on food prices*.

**Hypothesis 5 (H5).**  *Monetary expansion has a positive impact on food prices*.

**Hypothesis 6 (H6).**  *Energy prices have a positive impact on food prices*.

**Hypothesis 7 (H7).**  *Agricultural raw material imports have a negative impact on food prices*.

**Hypothesis 8 (H8).**  *Temperature level has a positive impact on food prices*.

**Hypothesis 9 (H9).**  *The global food price index has a positive impact on food prices*.

**Hypothesis 10 (H10).**  *The agricultural price index has a positive impact on food prices*.

**Hypothesis 11 (H11).**  *The fertilizer price index has a positive impact on food prices*.

**Table 1:** Summary of the literature.

Authors	Sample	Period	Method	Impact on Food Prices
Gilbert [32]	Global	1971–2008	Granger Causality	GDP → Food Prices, Exchange Rate (M) Oil Prices (M), Money Supply (M) Interests → Food Prices
Ahsan et al. [35]	Pakistan	1970–2008	ARDL	GDP per Capita (−), Subsidy (−) Money Supply (+), Food Crop (−) Global Food Prices (+), Agricultural Output (−)
Arango et al. [45]	United States	1960–2006	Panel Data Methods	Interest Rate (−)
Irz et al. [33]	Finland	1995–2010	VECM	Agriculture Commodity Prices (+) Energy Prices (+)
Joiya and Shahzad [44]	Pakistan	1972–2010	ARDL	GDP (+), Food Import (−), Food Export (+) Total Credit for Agricultural Sector (−)
Ahmed and Singla [38]	India	2006M1–2012M12	Johansen Cointegration Analysis	Exchange Rate (+), Energy Prices (+), Money Supply (+), World Food Prices (−), Rainfall (−), Interest Rate (−)
Bellemare [5]	Global International Data	1990–2011	Ordinary Least Squares	Rises in food prices foster social unrest
Sujithan et al. [40]	Global	2001/1–2013/3	Bayesian multivariate framework	Real Economic Activity (−), Biofuel Production (−) Oil Prices (−), Financial Markets (+)
Ismaya et al. [39]	Indonesia	2008: Q1– 2017: Q4	Generalized Method of Moments	Money Supply (+), Energy Prices (+), Food Production (−)
Hilegebrial [36]	Ethiopia	1971–2013	Ordinary Least Squares	Money Supply (M), Real GDP (−) Inflation Expectation (+) Global Food Price (+)
Rehman and Khan [37]	Pakistan	1990–201	VECM	Indirect Taxes (+), Food Export (+) Government Subsidy (−), GDP (−)
Fasanya and Olawepo [42]	Nigeria	1997/1–2017/4	Dynamic Conditional Correlation	Lending Rate (+), Exchange Rate (−) Oil Prices (+)
Kartal and Depren [41]	Türkiye	2004/1–2021/6	WC, QQ, GQ, QR	Fertilizer Price Index (+), Oil Prices (+)
Oral et al. [34]	Türkiye	2003/1–2022/3	Structural Vector Autoregressive	Money Supply (+), Oil Prices (+) Global Fertilizer Price Index (+), Global Food Price (+)
Ulussever et al. [43]	Global	1991/1–2021/5	ML Algorithms	Fertilizer Price (+), Raw Material Price (+)

Note: +: Positive Impact; −: Negative Impact; M: Mixed Results; →: Univariate Causal Impact.

## 3. Methods

To empirically examine heatflation in Nigeria, we used the most recent accessible data pertaining to the period between 2008/5 and 2020/12. In line with this objective, in this study, we considered Nigeria’s domestic food prices as the dependent variable and average temperature as the main explanatory variable, and we also incorporated various influential regressors. Thus, we gathered data from various sources, and Table 2 summarizes the details of all variables.

As is known, non-linearity and non-normality can easily occur in economic data [49], and one can obtain biased estimation results if these factors are ignored. In this regard, quantile-based empirical methods, namely, QQ, GQ, and QR methods, have been utilized to estimate the interaction between endogenous and exogenous variables. Quantile-based methods perform better amidst the existence of non-linearity in variables. Moreover, they are able to simultaneously investigate the nature of the existing interaction between variables in different quantiles [50]. In addition, the comparative use of QQ and QR allows researchers to check the consistency and robustness of their empirical findings. 

**Figure 2:**
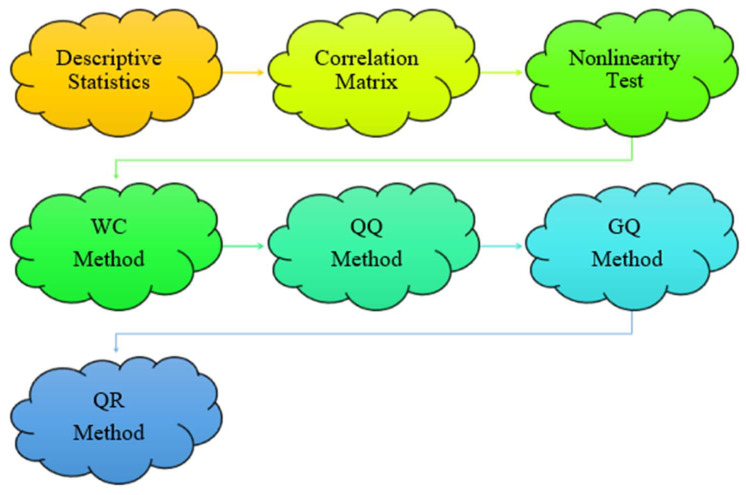
Empirical approach.

Moreover, the WC method can provide estimation results by simultaneously considering the time and frequency characteristics of the dependent and independent variables. Considering the frequency and time characteristics of the data can provide extended findings on the nature of the interaction. In line with main the aim of this study, a seven-stage methodology, illustrated in Figure 2, was used. First, the main descriptive statistics of the variables were determined. Second, the correlations between the variables were examined. Third, the non-linearities of the variables were analyzed using the BDS test [51], as this point, together with the normality distribution, is important for method selection. In the fourth step, the WC method [52] was applied to test the lead–lag nexus between food prices and explanatory variables. In the fifth step, the QQ method [53] was used to uncover the impacts of the explanatory variables on food prices across various quantiles. In the sixth step, the GQ method [54] was applied to investigate the causality nexus across the quantiles of the variables. Finally, the QR method [55] was applied to test robustness.

## 4. Results

### 4.1. Descriptive Statistics

As part of our empirical analysis, the main statistics of the variables were examined first. Table 3 summarizes the most important statistics.

Table 3 shows that Nigerian food prices fluctuate between 80 and 406 during the year, implying a spike in food prices. Among the other variables considered, food prices show the highest volatility. After that, fertilizer prices, oil prices, and agricultural prices show the highest fluctuations. In addition, all variables show a non-normal distribution, while the broad money supply shows a normal distribution. As stated by Pata and Yilanci [56], quantile-based methods provide effective results in the analysis of series with non-normal distribution; therefore, for this study, we used GQ and QQ approaches.

### 4.2. Correlation Matrix

In the second step of our empirical analysis, the correlations between food prices and explanatory variables were examined. Table 4 presents the correlations.

Table 4 shows that the explanatory variables correlate either positively or negatively with Nigerian food prices. On the one hand, all the variables except the prices of imported agricultural commodities and fertilizers have a positive correlation with food prices, ranging from 0.03 to 0.24. On the other hand, the three variables mentioned above have an overall negative correlation with food prices, fluctuating between 0.02 and 0.06. Additionally, there are some remarkable correlation levels in Table 4. There are relatively positive and high correlation levels between food exports, energy prices, global food prices, and agricultural price index. In addition, negative correlation values exist between temperature anomalies and cereal/crop yields and food production. It may be inferred that increasing temperature anomalies may be correlated with yield loss and food production, as was theoretically expected. These findings are consistent with our theoretical expectations. 

### 4.3. Linearity Test

In the third step, the non-linearity properties of the variables were investigated. Table 5 shows the results.

According to Table 5, all variables have a non-linear structure based on the BDS test. Overall, it can be seen that many of the variables have non-linear distributions, and all have a non-linear structure. Accordingly, we applied non-linear econometric methods (i.e., WC, QQ, GQ, and QR) for further empirical investigation.

### 4.4. WC Results

**Figure 3:**
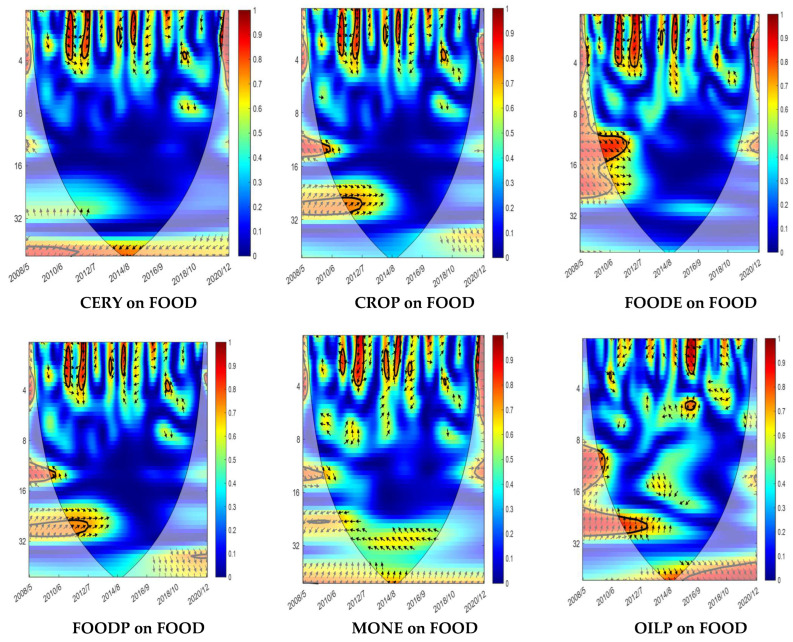

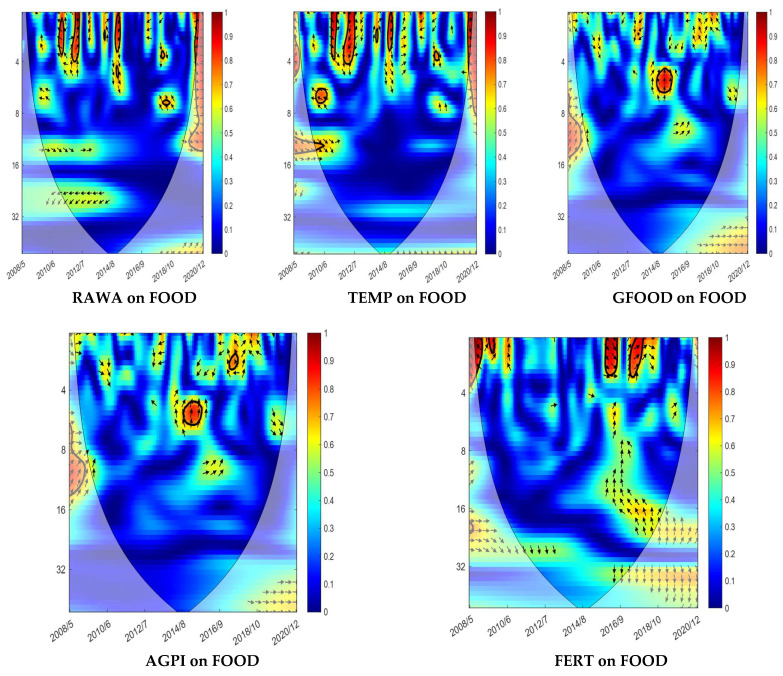
WC results.

In the fourth step, the WC method was used to analyze the nexus between Nigerian food prices and the explanatory indicators based on time- and frequency-varying structures. In all the WTC charts, “scale 0–8 indicates short term, scale 8–16 indicates medium term, scale 16–32 indicates long term, and scale 32–64 indicates very long term. 0–0.4 indicates low frequency, 0.4–0.6 indicates medium frequency and 0.6–1.0 indicates high frequency. The right arrows show a positive correlation while the left arrows show a negative correlation between the variables; right-down and left-up arrows show that the first variable causes the second variable, while right-up and left-down arrows indicate that the second variable causes the first variable. The first variable is FOOD (i.e., Nigerian food prices), while the second one is the relative explanatory variable” [52]. Figure 3 presents the results of the WC.

Figure 3 shows that CERY and CROP had either a positive or a negative strong nexus with FOOD in the short term around 2012/7 and 2014/8. In the long term, CROP also had a positive nexus around 2012/7. Thus, CERY and CROP have mainly a leading effect on FOOD, whereas FOOD lags behind.

FOODE had either a positive or a negative strong nexus with FOOD in the short term between 2012/7 and 2014/8. There was also a positive nexus in the medium term, around 2010/6. FOODE lags behind FOOD. In addition, FOODP had either a positive or a negative strong nexus with FOOD in the short term between 2010/6 and 2012/7. FOODP also had a positive long-term nexus around 2012/7. Thus, FOODP has a leading influence on FOOD.

MONE had either a positive or a negative strong nexus with FOOD in the short term from 2012/7 to 2016/9. In addition, MONE has a leading influence on FOOD, meaning that FOOD lags behind. OILP had a predominantly positive strong nexus with FOOD in the short term around 2016/9 and in the long term around 2012/7. However, RAWA had either a positive or negative strong short-term relationship with FOOD from 2010/6 to 2016/9, with RAWA having a leading influence on FOOD and FOOD lagging behind.

TEMP had either a positive or a negative strong nexus with FOOD in the short term between 2010/6 and 2014/9. TEMP also had a positive nexus around 2010/6 in the medium term. GFOOD and AGPI had a leading influence on FOOD in the short term between 2014/8 and 2016/9. Finally, FERT had a positive and negative strong nexus with FOOD in the short term between 2016/9 and 2018/10, with FERT leading and FOOD lagging.

The WC results show that there is a dependency between Nigerian food prices and explanatory indicators such as climate change (i.e., temperature) that varies according to time, frequency, and the explanatory variables.

### 4.5. QQ Results

**Figure 4:**
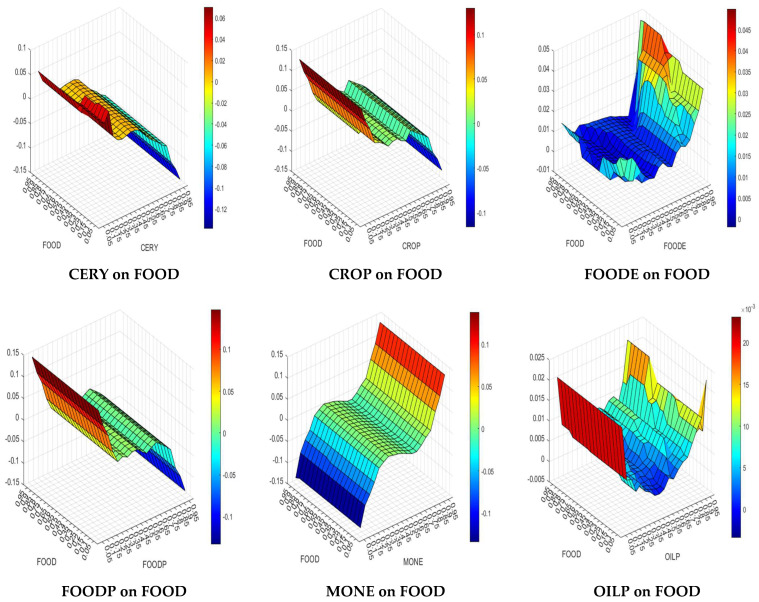

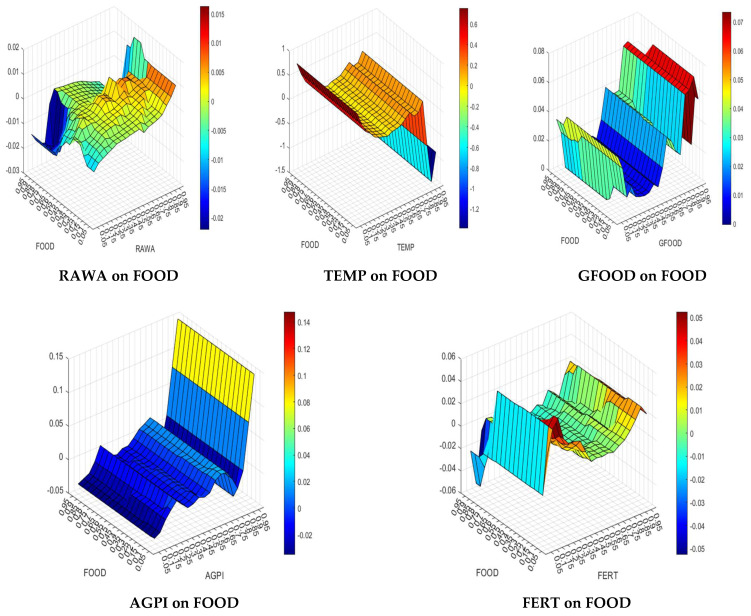
QQ results.

In the fifth step, the QQ method was applied to analyze the nexus between Nigerian food prices and the explanatory variables across the quantiles. Figure 4 presents the QQ results.

Figure 4 shows that the influence of CERY on FOOD increases at the lower quantiles. However, at the middle and higher quantiles (i.e., between 0.55 and 0.95), this influence decreases. Similarly, CROP has an increasing influence on FOOD at the lower and middle quantiles but decreases at the higher quantiles (i.e., 0.75–0.95). It can be said that the majority of the empirical outcomes are in favor of the acceptance of Hypotheses 1 and 2.

FOODE has an increasing influence on FOOD across all quantiles, whereby the increasing influence is stronger at the higher quantiles, implying that Hypothesis 3 is valid.

FOODP has an increasing effect at the lower quantiles, an almost insignificant increasing effect at the middle quantiles, and a decreasing effect at the higher quantiles (0.75–0.95). In contrast, an antithetical trend can be observed for MONE. MONE has a decreasing effect at the lower quantiles, an almost insignificant increasing effect at the middle quantiles, and an increasing effect at the higher quantiles (0.75–0.95). It can be said that the majority of empirical outcomes are in favor of the rejection of Hypothesis 4 and 5.

OILP and GFOOD have an increasing influence on FOOD across all quantiles, validating Hypotheses 6 and 9. However, RAWA has a decreasing effect at the lower quantiles, an almost insignificant increasing effect at the middle quantiles, and a decreasing effect at the higher quantiles. Thus, Hypothesis 7 can be considered valid. TEMP has an increasing effect at the lower quantiles, an almost insignificant increasing effect at the middle quantiles, and an increasing effect at the higher quantiles (0.85–0.95). Therefore, Hypothesis 8 can be considered valid.

Finally, AGPI has a decreasing effect at the lower quantiles, an almost insignificant increasing effect at the middle quantiles, and an increasing effect at the higher quantiles, rejecting Hypothesis 10. FERT has an increasing effect on FOOD across all quantiles, while the effect becomes almost insignificant and slightly decreases at the middle quantiles. It can be said that the majority of the empirical outcomes are in favor of the acceptance of Hypothesis 11.

Overall, the QQ results indicate that the effects of the explanatory indicators on Nigerian food prices change across the quantiles (levels), implying non-linear impacts.

### 4.6. GQ Results

In the sixth step, the GQ method was used to investigate the causal nexus between Nigerian food prices and the explanatory variables across all quantiles. Table 6 illustrates the GQ results.

Table 6 indicates that all variables have a causal influence on FOOD. However, the causal impact changes according to the quantiles. Specifically, the causal influence exists in all quantiles, except for some lower (0.05–0.10), middle (0.40), and higher (0.80–0.95) quantiles. It can therefore be concluded that the explanatory variables included in this study have an important influence on Nigerian food prices and can be used as predictors of Nigerian food prices.

### 4.7. Robustness Check 

Finally, the QR method was used to check consistency. A detailed comparison between the QQ and QR methods is presented in Appendix A, and the results are summarized in Table 7.

As Table 7 presents, all variables have higher consistency in predicting Nigerian food prices by QQ and QR methods, reaching ~99.99% for some variables. From this, it can be concluded that the results are consistent. Accordingly, policy caveats can be made based on the results.

## 5. Conclusions and Policy Caveats

### 5.1. Conclusions

In recent years, climate-related problems in the world have increased significantly. Due to the unintended consequences of global warming, environmental issues, and sustainable food supply have become the main concerns of countries and societies. Food is one of the main needs of humanity, and it is not possible to survive without having enough food. In this context, it is crucial to keep food prices stable so that all people have enough food to survive and grow healthily. Unfortunately, when temperatures rise in the world and in every country, many problems arise, such as droughts, which lead to decreases in food supply and, in turn, increases in food prices. Hence, it is critical to investigate the nexus between food prices and climate change in the context of heatflation. Accordingly, this study uncovered the heatflation risk for Nigeria, a leading country in the African continent where food-related problems are common. In doing so, this study analyzed Nigerian food prices, considering average temperature as the main explanatory variable and controls for a variety of different food-related factors, used monthly data from 2008/5 to 2020/12, and applied various novel non-linear methods for a comprehensive investigation.

The empirical results derived from the novel methods used show time-, frequency-, and quantile-based varying non-linear nexus between Nigerian food prices and temperature. The same is true for most of the other explanatory variables. Many explanatory variables even have an inverse effect (i.e., either from negative to positive or from positive to negative) after exceeding certain values across the quantiles. In addition, the explanatory variables were deemed to have causal influences on Nigerian food prices across the quantiles, with the causal effects varying for each pair of variables and across the quantiles.

To summarize, this study demonstrates the non-linear impact of temperature on Nigerian food prices, implying a heatflation risk. The magnitude of the effects and the causal impacts differs based on time, frequency, quantiles, and each explanatory variable in the context of controlling food prices in Nigeria. It should be noted that the results of this study generally align with the results presented in [34,36], but this study extends the current knowledge by providing more comprehensive details in examining the relationship between food prices and temperature in the context of heatflation.

### 5.2. Policy Caveats

Empirical evidence shows that temperature spikes and anomalies usually drive-up Nigerian food prices. One could say that there is a heatflation risk in Nigeria. Therefore, Nigerian governments should immediately focus on controlling the negative effects of global warming on food prices. To this end, policymakers should pay attention to increasing Nigeria’s adaptive capacity to climate hazards in alignment with the SDGs. In addition, the main policy option is to change Nigeria’s energy mix, which is heavily dependent on fossil resources [57]. To this end, Nigerian authorities should focus on expanding the country’s clean energy production capacity to reduce overall anthropogenic emissions and per capita emissions. The International Renewable Energy Agency (IRENA) has reported that the share of modern renewable energy sources in final energy was 1% in 2015, and it is hoped that this number will reach 8% and 10% by 2030 and 2050, respectively. Nigeria has an average annual global horizontal irradiation of 1600 kilowatt hours/meter for solar energy, while it has moderate wind potential, with average wind speeds at 10 m/h. The IRENA estimates of the technical potential for solar photovoltaic, wind, and hydro energy in the country are 210, 3.2, and 27.5 gigawatts, respectively. However, capacity utilization on solar, wind, and hydro energy was 0.017, 0, and 2.5, respectively in 2015 [57]. In this regard, the existing data reveal that Nigeria has the potential to combat climate change by deploying new renewable energy strategies, and establishing policies centered around the extension of renewable energy utilization is rational. In addition, Nigerian authorities should focus on improving the adaptive capacity of Nigerian agriculture to extreme weather events. In this regard, increasing research and development budgets for the development of climate-resilient agricultural technologies, restructuring cropping patterns and seasons to avoid wasting resources in climatically vulnerable regions, and investing in the transfer of knowledge regarding new technologies (e.g., hydroponic and vertical farming) could be effective policy options to balance Nigeria’s food supply and demand.

Considering that an increase in cereal yields and crop production has a declining impact on food prices, Nigerian policymakers should focus on strategies to increase cereal yields and crop production. However, to prevent increasing environmental pollution while increasing Nigerian cereal yields and crop production, the need for extra energy inputs should be provided by exploring the aforementioned renewable energy options. Thus, the possible negative effects of utilizing traditional energy sources could be prevented. Additionally, to increase the efficient use of Nigerian water in increasing food production, pollution prevention measures should be taken to improve the quality of Nigeria’s farmland and water systems. To this end, pollution taxes to maintain soil and water quality and prevent the waste of water could be effective policy options. In addition, an increase in the investment budget for Nigerian agriculture could increase the volume of agricultural production. In this way, the Nigerian food industry and farmers could benefit from the positive externalities of scale economics. Moreover, the use of innovative agricultural techniques is critical to increasing food production and reducing the risk of food shortages. Improving the technological infrastructure of the Nigerian agricultural sector could therefore increase the productivity and yield of grain and crop production. Therefore, Nigerian policymakers could consider a subsidy program for the technological transformation of Nigerian agriculture to improve crop yields and food and crop production, and tax exemptions for agricultural investments could be another policy tool. The supply side of the Nigerian food industry could be strengthened, the rise in food prices could be prevented, and the possible negative externalities of increasing food production by traditional techniques could be hindered. 

Considering the diminishing impact of agricultural raw material imports, which are vital for food production and impact food prices in Nigeria, as well as the increasing impact of food exportation on food prices, it could make more sense to encourage the importation of agricultural materials while curtailing the exportation of food products. Reducing tariffs on imports of agricultural products could therefore help to reduce food prices in Nigeria by boosting the supply of food. In addition, an increase in tariffs on food exports could encourage the Nigerian food industry to increase food production for the domestic market. To eliminate the impact of oil price spikes on price inflation, the Nigerian government could consider reducing the share of oil in the overall energy mix to prevent the negative impact of oil price hikes on food prices. In this context, replacing oil consumption with eco-friendly sources could be an option. Therefore, Nigerian policymakers could consider increasing government budgets to support green investments and subsidies for the private sector.

Nigerian food markets are sensitive to international events because there is transitivity between increases in global food and agricultural prices, global fertilizer prices, and Nigerian food prices. To reduce the impact of fluctuations in global commodity markets on Nigerian food markets, the relevant authorities could consider improving Nigeria’s storage infrastructure. The combination of developed storage technologies and an efficient inventory management system could mitigate the unintended impacts of global fluctuations on Nigerian food markets by increasing Nigerian food stocks and those of other agricultural commodities in times of abundance while releasing them to the market in times of scarcity and uncertainty. Therefore, the Nigerian government should focus on increasing the stock of physical and human capital in the relevant sectors.

An increase in money supply increases food prices in Nigeria. Therefore, decisions on quantitative easing should be taken deliberately. In this regard, linking money supply growth to economic growth could be an effective strategy for the Nigerian monetary authority. In this way, risky monetary expansions could be curbed, and the driving effect of excessive money on food prices could be eliminated. Thus, Nigerian individuals’ purchasing power can be maintained through quantity easing based on economic growth performance.

In summary, to maintain Nigerian individuals’ purchasing power while decisionmakers address the risk of heatflation and spikes in food prices, the aforementioned supply-enhancing policies could enhance the supply side of the food industry in Nigeria. Moreover, adopting a monetary policy by considering economic growth performance could help to stabilize the demand side of food industry and maintain the purchasing power of individuals by hindering unintended quantity cuts or eases that could heavily impact the purchasing power of individuals through recessionist or inflationist pressures. 

### 5.3. Future Directions

This study took a highly comprehensive approach to examining the impact of Nigerian food prices and a number of explanatory variables, particularly temperature. Although a good effort was made, there are also some limitations to this study. If these limitations are addressed, studies conducted in the near future may have a much broader context to uncover heatflation risks.

This study focused only on Nigerian cases. Although Nigeria is an important emerging African country, there are some other important countries (e.g., Türkiye) that have higher food prices. Therefore, future studies could examine these countries via empirical analyses. Even a comparative analysis for these countries, as well as for other groupings, such as higher and lower income countries, countries in the same risk groups (e.g., BRICS, E7, Fragile Five), and countries on different continents, could be conducted. Future studies could also consider extending the scope of their analyses by establishing predictive methods on the obtained findings by using ARCH-GARCH, dynamic ARDL, linear probability, wavelet local multiple correlation, and cross-quantilogram methods.

Lastly, although novel non-linear methods were used in this study to perform a time-, frequency-, and quantile-based analysis, these methods do not account for smooth structural shifts in the variables or interdependencies between the variables. Accordingly, future studies could apply Fourier-based estimators in further empirical analyses. The heatflation concept could thus be further explored by considering these aspects in future studies.

## Figures and Tables

**Table 2:** Variables.

Symbol	Explanation	Source
FOOD	Nigeria Food Price Index *	[46]
CERY	Cereal yield (kg per hectare)	[18]
CROP	Crop production index (2014–2016 = 100)	
FOODE	Food exports (% of merchandise exports)	
FOODP	Food production index (2014–2016 = 100)	
MONE	Broad money (% of GDP)	
OILP	WTI (Oil Price Index)	[47]
RAWA	Agricultural raw materials imports (% of merchandise imports)	[48]
TEMP	Average temperature	[12]
GFOOD	Food price index	[46]
AGPI	Agriculture price index	[48]
FERT	Fertilizer price index	

Note: * denotes the dependent variable.

**Table 3:** Descriptive statistics.

Variable	Mean	Median	Min.	Max.	Std. Dev.	Skewness	Kurtosis	Jarque–Bera	Probability
FOOD	191.48	164.50	80.80	406.40	86.43	0.75	2.43	16.25	0.0003
CERY	128.56	130.46	100.74	145.61	11.90	−0.78	2.72	15.96	0.0003
CROP	7.91	8.04	6.13	9.42	1.05	−0.12	1.56	13.52	0.0012
FOODE	0.24	0.20	0.06	0.49	0.11	0.81	2.38	19.22	0.0001
FOODP	7.92	8.08	6.29	9.34	0.98	−0.11	1.53	13.97	0.0009
MONE	2.02	2.05	1.75	2.32	0.14	−0.13	2.47	2.26	0.3237
OILP	70.32	67.97	16.55	133.88	24.26	0.29	2.22	6.00	0.0499
RAWA	0.10	0.07	0.02	0.37	0.09	2.02	5.86	155.48	0.0000
TEMP	2.29	2.29	2.27	2.32	0.02	0.47	1.99	12.19	0.0023
GFOOD	107.40	103.84	85.04	132.80	11.52	0.40	2.03	10.06	0.0065
AGPI	107.57	103.69	85.62	140.23	12.05	0.60	2.52	10.54	0.0051
FERT	139.67	127.47	88.14	342.68	48.42	1.83	7.36	204.86	0.0000

**Table 4:** Pearson correlation matrix.

	FOOD	CERY	CROP	FOODE	FOODP	MONE	OILP	RAWA	TEMP	GFOOD	AGPI	FERT
FOOD	1.00											
CERY	0.03	1.00										
CROP	0.03	0.78	1.00									
FOODE	0.24	−0.12	−0.18	1.00								
FOODP	0.03	0.78	0.99	−0.18	1.00							
MONE	0.00	0.08	−0.25	0.18	−0.25	1.00						
OILP	0.17	−0.01	0.06	−0.03	0.06	−0.06	1.00					
RAWA	−0.06	−0.77	−0.81	−0.03	−0.81	0.23	−0.02	1.00				
TEMP	0.05	−0.50	−0.44	0.59	−0.43	−0.05	−0.10	0.30	1.00			
GFOOD	0.13	−0.01	0.08	−0.10	0.07	−0.04	0.50	0.02	−0.15	1.00		
AGPI	0.15	−0.03	0.06	−0.11	0.06	−0.05	0.50	0.04	−0.15	0.99	1.00	
FERT	−0.02	−0.02	0.03	0.11	0.03	−0.09	0.09	0.04	−0.02	0.12	0.14	1.00

**Table 5:** BDS non-linearity test results.

Variable	M2	M3	M4	M5	M6	Result
FOOD	0.0000	0.0000	0.0000	0.0000	0.0000	NL
CERY	0.0000	0.0000	0.0000	0.0000	0.0000	NL
CROP	0.0000	0.0000	0.0000	0.0000	0.0000	NL
FOODE	0.0000	0.0000	0.0000	0.0000	0.0000	NL
FOODP	0.0000	0.0000	0.0000	0.0000	0.0000	NL
MONE	0.0000	0.0000	0.0000	0.0000	0.0000	NL
OILP	0.0000	0.0000	0.0000	0.0000	0.0000	NL
RAWA	0.0000	0.0000	0.0000	0.0000	0.0000	NL
TEMP	0.0000	0.0000	0.0000	0.0000	0.0000	NL
GFOOD	0.0001	0.0002	0.0023	0.0020	0.0005	NL
AGPI	0.0000	0.0000	0.0000	0.0000	0.0000	NL
FERT	0.0000	0.0000	0.0000	0.0000	0.0000	NL

Note: M and NL denote dimension and non-linearity, respectively.

**Table 6:** GQ test results.

Path	0.05	0.10	0.15	0.20	0.25	0.30	0.35	0.40	0.45	0.50	0.55	0.60	0.65	0.70	0.75	0.80	0.85	0.90	0.95
CERY → FOOD	0.37	0.10	0.01	0.01	0.01	0.01	0.01	0.56	0.01	0.01	0.01	0.03	0.01	0.01	0.01	0.09	0.42	0.53	0.25
CROP → FOOD	0.37	0.10	0.01	0.01	0.01	0.01	0.01	0.56	0.01	0.01	0.01	0.03	0.01	0.01	0.01	0.09	0.42	0.53	0.25
FOODE → FOOD	0.37	0.10	0.01	0.01	0.01	0.01	0.01	0.61	0.01	0.01	0.01	0.03	0.01	0.01	0.01	0.09	0.49	0.53	0.25
FOODP → FOOD	0.37	0.10	0.01	0.01	0.01	0.01	0.01	0.56	0.01	0.01	0.01	0.03	0.01	0.01	0.01	0.09	0.42	0.53	0.25
MONE → FOOD	0.37	0.10	0.01	0.01	0.01	0.01	0.01	0.56	0.01	0.01	0.01	0.03	0.01	0.01	0.01	0.09	0.42	0.53	0.25
OILP → FOOD	0.37	0.10	0.02	0.01	0.01	0.01	0.01	0.56	0.01	0.01	0.01	0.03	0.01	0.01	0.01	0.09	0.44	0.49	0.25
RAWA → FOOD	0.37	0.10	0.01	0.01	0.01	0.01	0.01	0.65	0.01	0.01	0.03	0.03	0.01	0.01	0.01	0.07	0.45	0.52	0.26
TEMP → FOOD	0.37	0.10	0.01	0.01	0.01	0.01	0.01	0.56	0.01	0.01	0.01	0.03	0.01	0.01	0.01	0.09	0.42	0.53	0.25
GFOOD → FOOD	0.37	0.10	0.01	0.01	0.01	0.01	0.01	0.56	0.01	0.01	0.01	0.03	0.01	0.01	0.01	0.09	0.42	0.53	0.25
AGPI → FOOD	0.37	0.10	0.01	0.01	0.01	0.01	0.01	0.56	0.01	0.01	0.01	0.03	0.01	0.01	0.01	0.09	0.42	0.53	0.25
FERT → FOOD	0.37	0.10	0.01	0.01	0.01	0.01	0.01	0.56	0.01	0.01	0.01	0.03	0.01	0.01	0.01	0.09	0.42	0.53	0.25

**Table 7:** Robustness Summary.

Variables	Correlation (%)	Variables	Correlation (%)
CERY and FOOD	99.65	OILP and FOOD	91.64
CROP and FOOD	99.84	RAWA and FOOD	76.45
FOODE and FOOD	96.95	TEMP and FOOD	99.99
FOODP and FOOD	99.99	GFOOD and FOOD	98.86
MONE and FOOD	99.99	AGPI and FOOD	99.95
		FERT and FOOD	96.22

**Figure A1:**
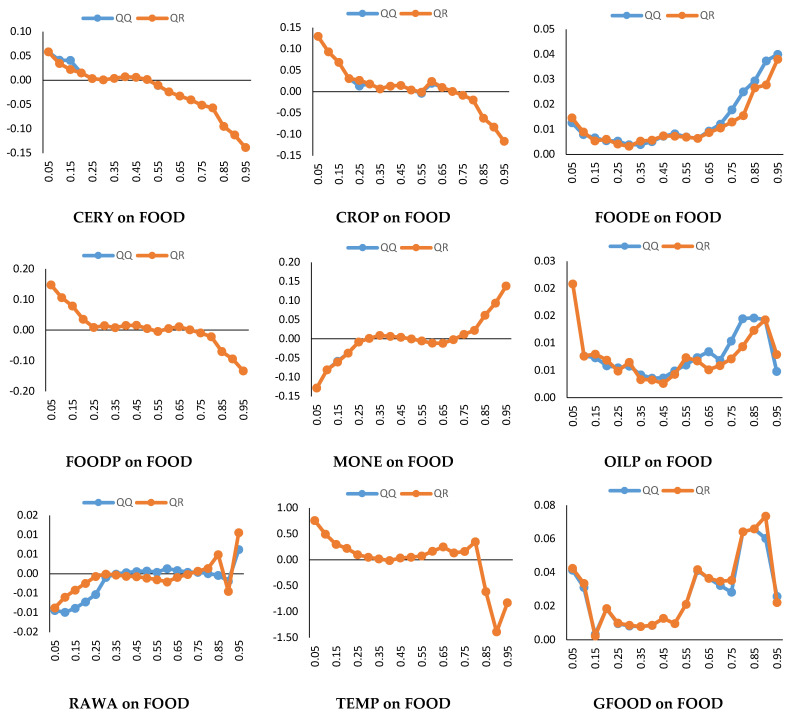

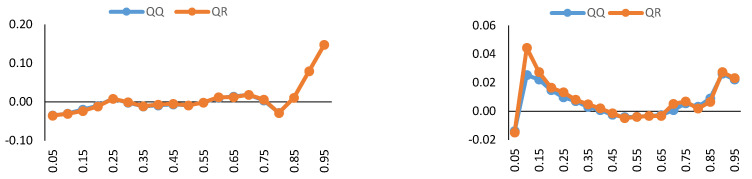
Robustness check.

## Data Availability

The data presented in this study are available on request from the corresponding author. The data are not publicly available due to locate in a file we organize.

## Abbreviations

ARDL	Autoregressive Distributed Lag
BDS	Broock, Scheinkman, Dechert, and LeBaron
DCC	Dynamic Conditional Correlation
GDP	Gross Domestic Product
GQ	Granger causality in quantiles
IMF	International Money Fund
IRENA	The International Renewable Energy Agency
ML	Machine learning
QQ	Quantile on quantile regression
QR	Quantile regression
SDGs	Sustainable Development Goals
TS	Time Series
VECM	Vector Error Correction Model
WC	Wavelet coherence
